# Managing clinical trials

**DOI:** 10.1186/1745-6215-11-78

**Published:** 2010-07-13

**Authors:** Barbara Farrell, Sara Kenyon, Haleema Shakur

**Affiliations:** 1 National Perinatal Epidemiology Unit CTU, University of Oxford, Oxford, UK; 2 School of Health and Population Sciences, University of Birmingham, Birmingham, UK; 3 Clinical Trials Unit, London School of Hygiene and Tropical Medicine, London, UK

## Abstract

Managing clinical trials, of whatever size and complexity, requires efficient trial management. Trials fail because tried and tested systems handed down through apprenticeships have not been documented, evaluated or published to guide new trialists starting out in this important field. For the past three decades, trialists have invented and reinvented the trial management wheel. We suggest that to improve the successful, timely delivery of important clinical trials for patient benefit, it is time to produce standard trial management guidelines and develop robust methods of evaluation.

## Introduction

Over the past 50 years, eminent trialists have written persuasively and repeatedly of the need for large, randomised, controlled trials [1], and such trials are considered the highest level of evidence for guiding clinical practice. However, how to manage these important trials has had little mention in most commentaries. Many clinical trials fail to deliver because of the lack of a structured, practical, businesslike approach to trial management. The human and financial resources for conducting a randomised trial is finite, so it is crucial that every effort is made to ensure that a trial is implemented simply and managed efficiently. A randomised trial involves a huge investment of time, money and people; therefore, it warrants expert management and needs to be managed from its inception like any other business. To review the literature, develop a protocol, apply for funding and design data collection forms requires lengthy consultations and a considered approach. Rarely is this essential depth of thinking applied to how the trial will actually be managed. Trial management is essential amongst the key competencies that are needed to deliver high-quality trials. It is recognised that well designed trials are the basis for addressing important clinical questions, but science alone will not be sufficient to successfully deliver a trial. Once the science is determined and the trial accepted through the peer review process, the challenge is quite different. The key challenge is then to establish and implement management systems and techniques that are effective and responsive to the needs of the trial and the trialist [2]. Clinical trials all require the same coordinated processes and systems, regardless of the size, scope, costs or duration.

An analysis of 114 multicentre trials funded by the National Institute of Health Research (NIHR) Health Technology Assessment (HTA) and UK Medical Research Council (MRC), STEPS [3], showed that 45% failed to reach 80% of the prespecified sample size. Less than one third of the trials recruited their original target number of participants within the time originally specified, and around one third had to be extended in time and resources. One factor observed in trials that recruited successfully was that they had employed a dedicated trial manager (odds ratio: 3.80, 95% CI: 0.79 to 36.14; *P *= 0.087). The STEPS collaborators suggest that anyone undertaking trials should think about the different needs at different phases in the life of a trial and put greater emphasis on 'conduct' (the process of actually doing trials) [3]. In addition, the MRC acknowledged that the failure of some trials can be due to practical problems with trial management rather than scientific problems or problems with the trial design [4]. Francis et al. [3] examined whether clinical trials could be considered from a business management perspective and proposed that the dimensions of running a successful trial includes 'marketing', 'sales' and 'ongoing client management'. They recognised that in the recruitment stage of a randomised controlled trial (RCT), the most demanding activity is to establish and implement a range of effective management techniques which parallel those used to run a successful business.

## What makes a successful trial?

Prescott et al. [5] assembled and classified a comprehensive bibliography of factors limiting the quality, number and progress of RCTs. They identified barriers to clinician participation that included, for example, time constraints, concern about the impact on doctor-patient relationships, concern for patients, lack of reward and recognition, and an insufficiently interesting question. Barriers to patient participation included issues such as additional demands of the trial, patient preferences, concern caused by uncertainty and concerns about information and consent. They recommended that to overcome barriers to participation, a trial should address an important research question and the protocol and data collection should be as straightforward as possible, with demands on clinicians and participants kept to a minimum. Dedicated research staff may be required to support clinical staff and participants. The recruitment processes of an RCT should be carefully planned and piloted regardless of size or complexity.

On the basis of experience in noncommercial academic initiated trials, Farrell and Kenyon [6] in *The Guide to Efficient Trial Management *suggest that actively managing every aspect of the trial is key to success. If clinicians are to recruit participants, they should feel comfortable and trained in trial processes and procedures. This can be achieved using a variety of methods: one-to-one training, group work, distance learning methods (videos via the web and teleconferences). National and international presentations and discussions to continually highlight the importance of the trial must be organised by the trial team. Maintaining a personal interface with a collaborative group of clinicians, whether this is a group of 7 or 700, is probably the biggest challenge for a trial manager and the trial team but one that will result in a more cohesive trial.

### A trial manager

The importance of a trial manager to the success of the project is recognised by the NIHR HTA programme, and they recommend that all primary research projects appoint a dedicated project/trial manager. Ideally, trial managers should be involved early on in the trial design phase, but this is rarely possible because of funding constraints. However, a good trial manager involved in the trial design and funding application will make a valuable contribution to the practicalities of conducting the trial, potentially saving money and avoiding unworkable systems. Generic job descriptions produced by the HTA [7] and the UK Trial Managers' Network (UKTMN) [8] identify the key responsibilities of a trial manager as follows:

• Having a leading role in planning, coordinating and completing a project

• Excellent communication and presentation skills

• The ability to organise and motivate others

• Flair, enthusiasm, innovation and leadership when faced with challenges

• The ability to manage the trial budget(s) and maintain the accounts

• Having strategic, tactical and operational management skills in the planning and execution of a project

Despite the complex responsibilities of this role, the body of knowledge available to guide trial managers is very limited. In 1998, Farrell [9] described the need for trial management models and methodology to be established, recognised and published to provide a body of evidence for those undertaking clinical trials, large or small; yet more than 10 years on very little recognised reference material, other than *The Guide to Efficient Trial Management *[6], is readily available. In addition to a trial manager, an efficient, well-trained trial management team can be the deciding factor in the success or failure of a trial. The trial team will be decided by the needs of the trial itself and, apart from the Chief Investigator, it should include a trial manager, a trial statistician, a trial programmer, a data manager, data clerks, administrative staff and other trial specific staff, e.g., health economists. Each of these has an important role to play, and clarity about exactly what each of the roles involves is crucial if every aspect of a trial is to be managed well; it requires a team effort. In the UK, the recent development of research networks and the registration of Clinical Trials Units has seen the 'portfolio trial manager' emerge. The portfolio manager has to deal with a variety of tasks across a range of trials which can present different challenges. The success or failure of this approach will become apparent over the coming decade.

### Project planning

A clinical trial shares many features with any other type of business project as defined in the field of project management [10]. These features include the following:

• A clear objective aimed to bring about change

• Requiring a team

• A set time scale

• Defined resources to achieve its objective

• Tasks which need to be completed (to a prespecified standard)

All projects consist of a series of processes, a set of actions to bring about results. The five basic process stages are [10]:

1. Initiating

2. Planning

3. Executing

4. Monitoring and controlling

5. Analysis and reporting

These five stages reflect the life cycle of a trial. Therefore, developing a management plan is key for effective trial management. It is essential that a project management plan include details of the arrangements for developing and monitoring all aspects of a trial, including servicing the steering committee and the independent data monitoring committee but, most important, how the day-to-day running of the trial will be planned and managed. The development of a robust statistical analysis plan supported with sufficient resources and time to conclude the trial efficiently is a crucial element of this plan. The project plan should also describe who will be responsible for essential activities, such as staff recruitment, staff management, communication with the collaborative group, recruitment monitoring, data management, and raising project awareness (promotion), through to safety reporting, analysis, report writing and dissemination of the trial results. The project plan should describe what the trialists are trying to achieve, how resources will be used and within what time frame. It should also include how the planned processes will be monitored to ensure that the project is being delivered as planned. The plan can then be reviewed and refined, if necessary, as the trial progresses. Clear processes, both inside and outside the office, need to be established and documented. The ability to constantly review and adapt the project plan is crucial as a trial can be hit side-on by events outside its control, e.g., emerging evidence, war leading to lack of recruitment and natural disasters. Sensible risk assessment, tailored quality assurance management systems and real-time monitoring are essential if a trial is to optimise its potential and provide reliable evidence.

The Clinical Trial Toolkit [11] was developed in 2003 to coincide with the implementation of the EU Clinical Trials Directive [12] by the UK Medical Research Council (MRC) and the UK Department of Health as a tool to guide people embarking on a clinical trial through the regulatory and governance requirements. The toolkit is a good starting point for trialists and trial managers to ensure all legal obligations are met, but it does not specify how to run a trial.

### Collaboration

Good evidence that the clinical question being evaluated is in equipoise is important, but it is only part of the equation. The question also needs to be relevant to clinicians and nurses as they are likely to be the people recruiting the participants. To be successful, most trials depend on developing some sort of collaborative group. The aim of a collaborative group or network is to be inclusive rather than exclusive. Proactively raising the profile of any developing project and creating a group of interested people takes time and commitment. This can be done in many ways, through personal contact, presentations at relevant conferences, mailshots, newsletters from the professional colleges, journal articles and general word of mouth. The success of a trial, particularly recruitment, may require thinking 'outside the box' and training, supporting and crediting other groups who are not traditionally directly involved in the research process but nevertheless are crucial to a trial: for example, nurses, records department staff, ward clerks, radiology staff. A trial is likely to be more successful, and enjoyable, if members of the collaborative group feel they 'own' the project. This ownership will be fostered by involvement and consultation at every stage, from protocol development to publication of the results. All trials need to be actively promoted or marketed. Part of this strategy will be a memorable name and/or identifiable logo and a thoroughly professional image. It is well established that interdisciplinary collaboration offers greater potential for success [13]. For large trials, this will be a diverse multidisciplinary group including representatives from each participating site. For smaller and single-centre studies, the group will be less formal and may be just a handful of like-minded people. Bammer [14] identified that there is a growing body of research on collaborations which include examinations of the increase in collaborations and team sizes, patterns of collaborative networks, motives, choices and strategies for collaboration, the measurement of collaboration, how collaborations are organised and how successful collaborations are measured. However, how these concepts are applied to trial management is unclear, and further observation and evaluation are necessary but difficult to carry out. The only reliable way to obtain good evidence that business concepts work in a clinical trial would be to conduct a randomised trial. One half of the trial would be managed according to a project plan and the other half left to run without a plan; we suspect this would be unacceptable to any funder and certainly unacceptable to a good trial team. Some elements of trial management may be easier to evaluate, such as the best method of ensuring data are completed and returned, but once again this carries an element of risk (as does any trial) and will have resource implications.

### Minimal work for investigators and participants

Minimal work for investigators and participants means ensuring recruitment procedures run alongside routine practices. Site visits and talking to staff in the place where recruitment happens will make sure recruitment to the trial becomes part of the daily routine. The recruitment procedure needs to be realistic and practical; for example, web randomisation may not be practical for a trial being conducted in an Intensive Care Unit or in a trial of an emergency intervention. Clinical staff are always busy and may be reluctant to carry out complex procedures to recruit participants. Complicated procedures and extra tests or visits may also deter the enthusiastic participant. The data that need to be collected to answer the clinical question should be readily available to the recruiting staff.

Development of the data collection forms should begin early in the process of trial development. Ideally, dummy tables that reflect the final analysis would be prepared as part of the statistical analysis plan to ensure that the data collection forms do not collect unnecessary data. This takes considerable discipline but will avoid omissions in the data collection forms and minimise the collection of data that will never be reported. Experience has generated some simple tips for the design of data forms such as always collect the raw data; if necessary, it can be put into categories later. Questions should be ordered in a way that reflects clinical progression and makes sense to the person completing the form. Data collected as 'free text' is not advisable, as this can considerably increase the data management workload and increase the risk of misinterpretation of the data, but is nevertheless sometimes unavoidable. A recent article by Edwards [15] provides a theoretical guideline for questionnaire design and administration but acknowledges that further evaluation is required.

### Communication

Investigators need to feel valued and part of an inclusive team answering an important clinical question, so providing regular feedback that ensures they feel involved must be central to a trial's communication strategy. Remembering the audience being addressed and tailoring all communication appropriately will help busy clinicians identify his or her priorities and maintain trial 'buy-in'. Using an investigator's preferred method of communication (telephone, email, letter, web site and personal contact) will ensure he or she feels communication is personal. Projecting a positive image about trial progress generally as well as progress within any given site will encourage continued involvement. Listening to problems and resolving any issues quickly will increase confidence in the trial and the trial team. Investigators should always be made to feel appreciated and not over burdened by involvement in the trial.

### Efficient systems

A trial, particularly a large trial, needs robust computerised systems and procedures that monitor every aspect of the day-to-day running of the trial. A reliable system that will monitor recruitment, randomisation procedures, stock control, data management, data cleaning, and central data monitoring and that will produce useful reports should be developed. Every essential piece of paper that relates to a trial participant should be logged and tracked through the system. There needs to be a logical and transparent structure, concise documentation (standard operating procedures) and accountability of every process employed in the trial. If the trial is international, these systems should take account of differing clinical practices, working environments and governance regulations.

Good quality data depend on effective trial management. Collecting data by the use of a case report form and entering it into a database are quite simple tasks. However, ensuring that these data are sensible, reliable and reflect the 'true situation' is a complicated and detailed process. With the aid of computers, data validation and quality control can be quick and efficient, but these systems also need to be flexible and adaptable so that they can respond to the needs of the investigators and the changing needs of the trial. Using systems that reduce the number of steps required for data entry, such as the use electronic data capture, can minimise the workload for both investigators and the data management team. However, if trialists intend to use electronic data capture, a good deal of preparatory work needs to go into form design and training to avoid adding to the workload. Adherence to database design, testing and validation standards is crucial during the computer system development process and required under clinical trials legislation [16].

### Efficient recruitment of trial participants

A trial succeeds or fails on the basis of whether it manages to recruit the prespecified number of participants to reliably answer the question, and yet there is very little research evidence to guide recruitment strategies. Mapstone et al. [17] identified 15 eligible trials aimed at recruiting participants for health care studies. Trials of monetary incentives, an additional questionnaire at invitation and treatment information on the consent form demonstrated benefit. However, these specific interventions from individual trials are not easily generalisable. The authors concluded that on the basis of this evidence, it is not possible to predict the effect most interventions will have on recruitment. A Cochrane review on incentives and disincentives to participation by clinicians in RCTs by Rendell et al. [18] found 11 relevant observational studies relating recruitment rates to a number of factors. In particular, these studies suggested that there was more recruitment if the clinician

• Was interested in evidence based practice

• Was participating in an academic group

• Had extra staff to help with recruitment

• Thought patients might be interested

• Felt comfortable about explaining trials

Although these may provide some pointers for areas to address, the review authors concluded that the research evidence base for strategies for increasing recruitment was poor and that further research was needed. Experienced trial managers, who have learnt through apprenticeship, continually monitor, review and revise the recruitment strategies being used, and this body of experience has been published online as part of *The Guide to Efficient Trial Management*[5]. To maintain recruitment at the necessary level over a long period of time, say, 3 to 5 years, requires stamina in everyone involved in a trial. Strategies used to do this might include visiting sites where the trial is working well and seeing what lessons can be learnt and applying them elsewhere. Using the experiences of individuals within the collaboration who are doing well to teach others, either in newsletters or at meetings, is very valuable and encourages internal collaboration and capacity building. Ensuring there is always clear, professional literature regarding the trial at recruiting sites is a task that a good trial manager will incorporate into the project plan. If promotional material is not updated regularly with new eye-catching information, it quickly becomes just part of a sea of other information and all impact is lost. Making sure the trial team go to meetings prepared, i.e., knowing how sites are recruiting, the quality of the data collection and who are the most important people to meet to discuss the trial's progress, should be a 'given', but this is not always the case.

### Publication and dissemination

How credit for the trial will be shared is also an important component of the project development and management plan. For collaborative trials, it is vital that appropriate credit is given where it is due and that everyone who has wholeheartedly contributed gains recognition in one way or another. This will often mean publication of the results as a collaborative group. Group authorship is a particular issue for trial managers as under the collaborative authorship policy rarely does the trial manager get acknowledged for their individual contribution. This is a real concern for those working toward making trial management their career pathway and those working to promote a career structure for trial managers.

The trial will mean nothing if the results are not disseminated and taken account of in clinical practice. Results of a trial can be made widely available using a variety of media, such as articles in medical journals, online journals, trial registers, systematic reviews and conference presentations. An advantage of a multicentre trial is that each investigator, working within an agreed policy, can be responsible for local dissemination and presentation. Trial results should be published whatever the outcome of the trial, and it has been described as scientific misconduct not to publish [19]. Reporting the results must maintain confidentiality, and it must not be possible to identify individual participants or sites within the report. The CONSORT Guidelines [20,21] provide a standard for reporting clinical trials which aims to improve the quality and transparency of trial reporting.

### Education, training and experience

The EU Clinical Trials Directive 2001 [12] specifies that every member of a trial team should have the appropriate education, training and experience to perform his or her tasks. For a trial manager of any trial, it is difficult to comply with this regulation as specialised training in trial management does not exist and there is no recognised qualification that can prove that a trial manager has been educated in the discipline. Much of the collective wisdom about doing trials has been passed on by apprenticeship, very much a 'suck it and see' approach which can be to the cost of the trial and the trial manager. For those wishing to pursue a career in trial management, the lack of good practice guidelines and standards can be extremely challenging and at times very frustrating. A survey undertaken by the UKTMN in 2005 identified the need for courses in practical management of clinical trials. Of the 284 trial managers surveyed, 60% were not seeking higher education qualifications but wanted flexible, accessible, specific training relevant to their jobs. For those who do want to study for a higher qualification, the distance learning MSc/Post Graduate Diploma in Clinical Trials by Distance Learning [22] has been developed by the London School of Hygiene and Tropical Medicine and the University of London. The MSc includes project management principles as one of the fundamentals of trial conduct. Many other courses and workshops on clinical trials also include an element of project management skills training. However, although intuitively trial managers are utilising these skills, more evidence is needed to support the application of project management principles and practices to clinical trial management for future trialists.

## Discussion

A recurring theme in this paper is the need for those planning and doing trials to have reliable and rapid access to relevant expertise and for published standards for trial management (conduct) that avoid trialists' reinventing the wheel. Trial managers have, in recent years, begun to develop better ways of disseminating and sharing experiences and expertise. Societies and associations of trial managers in North America and Europe are beginning to network and make their knowledge available via the Internet and through journal publications. The acknowledgment by the UK Medical Research Council in setting up the UK Trial Managers' Network in 1998 highlighted the need to share expertise in this field and to bring together trial managers who have no professional forum in which to network. Experienced trialists will have put together, either formally or informally, plans and checklists of essential steps in the development of a trial on the basis of their experience of what does or does not work. Those planning their first trial often have to start from scratch unless they are lucky enough to have access to a clinical trials unit or someone with relevant experience. Many trials struggle to finish, or even to get underway, because the people running them have not been able to find information about the best processes for establishing and delivering a trial. There is a need for appropriate training which is easily accessible, but the real problem is the lack of a standard method which will ensure high-quality trial management. Having such a standard would ensure that both funders and trial managers maximise the trial investment and the chances of success. Much of trial management is intuitive utilisation of skills gained in other areas of work or on the basis of experience and as such could not be the subject of robust research methodologies. Robust, meaningful and enforceable standards for the management of trials would require effort on behalf of a collaborative group (using trial management principles), including funders, investigators, trial managers and other interested groups and would take time but would do much to move the issue forward. If such a standard could be agreed on, this would more accurately identify training requirements and open the door to more appropriate research into what is undoubtedly a vital component in successfully completing a clinical trial.

## Conclusion

The very important and internationally accepted CONSORT Guidelines were developed because there was a will to improve the way important research was published. There is the same will amongst trialists to improve trial management methods and provide sound published evidence to be used to successfully evaluate important health research. We urge that funders, trialists, trial managers and all interested groups come together, led by opinion leaders in the field, to discuss and debate trial management methods with the aim of providing a standard for trial management and a guideline for those running clinical trials to work toward. We also suggest that the editors of medical journals might want to consider the importance of how good research is actually carried out and require that trial management methods be part of articles considered for publication. If trial management continues to be unrecognised through a lack of standard methodology and training, it will be to the detriment of future research and health care.

## Competing interests

The authors declare that they have no competing interests.

## Authors' contributions

All authors contributed to the development and production of the manuscript. All authors read and approved the final manuscript.
